# Multidisciplinary Group Education for Gestational Diabetes Mellitus: A Prospective Observational Cohort Study

**DOI:** 10.3390/jcm9020509

**Published:** 2020-02-13

**Authors:** Caro Minschart, Kelly Amuli, Anouk Delameillieure, Peggy Calewaert, Chantal Mathieu, Katrien Benhalima

**Affiliations:** 1 Department of Endocrinology, University Hospital Gasthuisberg, Catholic University Leuven, Leuven 3000, Belgiumchantal.mathieu@uzleuven.be (C.M.);; 2 Nursing and Midwifery Research Group, Brussel Health Campus, University Hospital Brussel, Brussels 1000, Belgium; 3 Department of Chronic Diseases, Metabolism and Ageing, Laboratory of Respiratory Diseases, Catholic University Leuven, Leuven 3000, Belgium

**Keywords:** gestational diabetes mellitus, treatment, education, group education

## Abstract

The value of diabetes education, focusing on lifestyle measures, in women with gestational diabetes mellitus (GDM) is acknowledged, but requires intensive education and input of resources if done on an individual basis. Group education could be a valuable alternative to individual education. This study aims to investigate the impact of multidisciplinary group education on women’s knowledge about GDM, education, treatment satisfaction, and emotional status. Two hundred women with GDM were enrolled in a prospective observational study. Dutch speaking women were offered group education at their first visit after GDM diagnosis. Non-Dutch speaking women or women for whom group education was not possible received individual education. Individual follow-up with a dietitian was planned within two weeks for all women. Women receiving individual education (*n* = 100) were more often from an ethnic minority background compared to women in group education (*n* = 100) (32.0% (*n* = 31) vs. 15.3% (*n* = 15), *p* = 0.01). Knowledge about GDM significantly improved after education, with few differences between the two education settings. Both patients in group and individual education were equally satisfied with the content and duration of the initial and follow-up education. Of all group participants, 91.8% (*n* = 90) were satisfied with group size (on average three participants) and 76.5% (*n* = 75) found that group education fulfilled their expectations. In conclusion, women diagnosed with GDM were overall satisfied with the education session’s content leading to a better understanding of their condition, independent of the education setting. Group education is a valuable alternative to better manage the increasing workload and is perceived as an added value by GDM patients.

## 1. Introduction

Gestational diabetes mellitus (GDM) is one of the most frequent medical conditions during pregnancy and is defined as diabetes diagnosed in the second or third trimester of pregnancy, provided that overt diabetes early in pregnancy has been excluded [1]. GDM is associated with an increased risk for fetal and maternal complications such as preeclampsia and macrosomia [2,3]. In the long term, women with GDM have a seven-fold increased risk of developing type 2 diabetes mellitus (T2DM). The offspring is also at increased risk of developing obesity, metabolic syndrome, and T2DM [4,5,6,7,8]. The ‘International Association of Diabetes and Pregnancy Study Groups’ (IADPSG) and the World Health Organization (WHO) currently both recommend a universal one-step strategy with a 75 g oral glucose tolerance test (OGTT) for the screening of GDM [9,10]. As these diagnostic cut-off values are more stringent and one abnormal glucose value is sufficient for the diagnosis of GDM, adoption of the new IADPSG criteria leads to an important increase in the prevalence of GDM, creating an important increase in workload and associated costs [11].

Treatment of women with GDM results in a lesser degree of perinatal complications and can potentially improve the health-related quality of life [2,3,12,13]. Initial treatment of GDM involves non-pharmacological approaches such as medical nutrition therapy, weight management, physical activity, and glucose monitoring [1]. If lifestyle measures are insufficient to reach and maintain glycemic targets, pharmacological therapy should be added [1]. Treatment of GDM should always start with education about medical nutrition therapy, physical activity, weight management, and self-monitoring of blood glucose (SMBG). The concept of self-management is thereby crucial to achieve good maternal and neonatal outcomes [14]. Therefore, diabetes educational programs for women with GDM should be organized in order to help them cope with their condition during pregnancy. However, the management of GDM is a labor-intensive discipline in which the rapidly rising prevalence of GDM poses challenges to maintain high-quality care [11].

Group education is a well-documented alternative to individual education in the organization of diabetes care in general, serving as a method to meet the educational needs of diabetes patients while at the same time providing peer support and motivation [15,16]. However, few studies have investigated the effectiveness of group education for the treatment of GDM. A study investigating multidisciplinary group education in women with GDM found that group sessions were associated with a reduction in carbohydrate consumption, an increase in physical activity level, and a combined clinical time saving of 8–28 h per week [17]. Another observational study in the United States demonstrated that women with GDM in group prenatal care required less insulin treatment, attended post-partum follow-ups more often, and underwent more often postpartum glucose screening compared to women who received conventional obstetrical care [18]. Factors associated with group education such as learning from the experience of peers, a greater connection with health care providers and a motivating group dynamic may partially explain these beneficial results [18]. A recent study demonstrated the benefits of group education sessions delivered by a specialized diabetes midwife and a dietitian on women’s knowledge of GDM, but made no comparison with individual education sessions [19]. Additional high-quality studies in this research area are necessary to evaluate the feasibility and impact of group education compared with conventional individual education sessions in the management of GDM. This study aimed therefore to determine the impact of a multidisciplinary group education program for the management of GDM on women’s knowledge about GDM, their satisfaction about the education and treatment, and their emotional status.

## 2. Materials and Methods 

This study was performed in compliance with the principles of the Declaration of Helsinki (2008) and received approval from the local Ethics Committee of UZ Leuven (B322201525589). Prior to the first inclusion, the study was registered in Clinicaltrials.gov (NCT02528162). Participants provided written informed consent before inclusion in the study.

### 2.1. Study Design

This monocentric prospective observational cohort study was conducted at the University Hospital UZ Leuven in Belgium from October 2015 to September 2018. Since October 2015, the endocrinology department of UZ Leuven replaced the initial individual education session for the management of GDM as much as possible by structured group education sessions. Screening for GDM was based on a universal two-step screening strategy with a 50 g glucose challenge test (GCT) and a 75 g oral glucose tolerance test (OGTT) using the IADPSG/2013 WHO criteria. After diagnosis of GDM, Dutch speaking women were invited to attend a multidisciplinary group education session of maximum 1.5 h with a maximum of six participants. The session was organized on a weekly basis and was provided by a certified diabetes educator and a specialized diabetes dietitian. For non-Dutch speaking women or if group education was not possible, the first education session was delivered individually. A structured PowerPoint presentation was used both in the group and individual education sessions to educate about the pathophysiology, consequences and treatment of GDM—including dietary intake, physical activity and SMBG. The structure and content of this presentation was evaluated on a regular basis and adapted if necessary to the most recent guidelines and recommendations. For non-Dutch speaking patients, the presentation was translated to French and English. At the end of the education session, women received the handouts of the presentation, a brochure with information on physical activity, a glucose monitoring diary, a seven-day diet journal, a brochure on specific dietary guidelines with adapted recipes and material for SMBG.

Regardless of the initial education setting, all women were offered an individual follow-up session of 30 min with a dietitian within two weeks after the initial education session. During this session, women received further advice regarding their gestational weight gain and dietary habits based on their seven-day diet journal together with the SMBG results. The glycemic targets of the American Diabetes Association (ADA) were followed (fasting plasma glucose < 95 mg/dL (5.3 mmol/L) and two hours after meals < 120 mg/dL (6.6 mmol/L)) [1]. Further follow-up of glycemic values occurred every two weeks through email, phone or by attending the diabetes outpatient clinic as needed. In case of persistent inadequate glycemic control, treatment with insulin was started in consultation with an endocrinologist and women were followed-up at the outpatient diabetes clinic every two weeks until delivery. As part of normal routine, women with GDM were offered a 75 g OGTT three months after delivery to screen for glucose intolerance according to the ADA criteria [1]. Treatment and follow-up of women participating in the study were in line with normal routine for the management of GDM. There were no additional medical interventions, extra visits or additional blood tests compared to the treatment of women who did not participate in the study.

### 2.2. Study Participants

Women diagnosed with GDM could participate if at least 18 years old. Women were excluded if they had a history of bariatric surgery, were diagnosed with pregestational diabetes or if they could not speak fluently Dutch, French or English. All other women attending a group or individual education session were invited to participate in the study. 

### 2.3. Study Assessments

Data were collected from the electronic medical records and through questionnaires. Outcome data from women who received the initial education session in group were compared to those from women who received the initial education session individually.

#### 2.3.1. Self-Administered Questionnaires

All participants—both in group and individual education—were asked to complete several questionnaires at three different time points during the multidisciplinary education program: prior to the initial education session, immediately after the initial education session and after the individual follow-up session with the dietitian. The questionnaires aimed to evaluate the education of GDM, the knowledge on GDM and the emotional status. For this purpose, the questionnaires measured sociodemographic characteristics of the subjects, knowledge of GDM, feelings of depression and anxiety associated with the diagnosis of GDM, and the satisfaction with the education and the treatment. It took about 10 to 15 min at each point in time to fill in the questionnaires. All questionnaires were translated into French and English for non-Dutch speaking participants. An overview of the questionnaires administered at each time point is given in Table 1.

Questionnaire I on sociodemographic characteristics: A self-designed sociodemographic questionnaire—including data on education level, ethnicity, financial and marital status—was administered at the start of the initial education session. This questionnaire was based on a questionnaire that has previously been used in the Belgian Diabetes in Pregnancy Study [20].

Questionnaire II on knowledge of GDM: Knowledge of GDM was assessed before and after the initial session and after the follow-up session to measure knowledge gains, using a self-designed questionnaire containing 14 multiple-choice questions on risk factors for and consequences of GDM, diagnosis of GDM, treatment of GDM and follow-up after delivery. Proportions of correct responses on the knowledge questionnaire of the total cohort prior to the initial education session were compared to those after the follow-up session in order to evaluate knowledge improvement after education. In order to compare the knowledge about GDM between participants in group education and those in individual education, response rates on the knowledge questionnaire after the initial education session were compared between both groups. 

Questionnaire IIIa and IIIb on satisfaction with the education sessions: Two self-designed questionnaires were created to evaluate satisfaction with the education program and whether treatment goals could be achieved. The first education satisfaction questionnaire (IIIa) was administered after the initial education session and evaluated the participant’s degree of agreement with the clarity and relevance of the explanation on twelve items that were discussed during the presentation, using a five-point Likert scale. This questionnaire also contained an extra section with multiple choice questions for women attending the group education to evaluate perceptions on the duration of the group session, the advantages and disadvantages of group education and the size of the group. At the end of the questionnaire, an open question was included to share comments about the education session. The second education satisfaction questionnaire (IIIb) was completed after the follow-up session with the dietitian and again assessed participant’s satisfaction with the twelve discussed items during the session. An additional set of questions on a five-point Likert scale was included regarding patient satisfaction on accomplishing lifestyle modifications in the week following the initial education session. At the end of this questionnaire, women were given the opportunity to share their opinions about the education session in an open question. 

Questionnaire IV on depression: To measure possible feelings of depression, the ‘Center for Epidemiologic Studies Depression’ (CES-D) questionnaire was completed at all three time points. The CES-D questionnaire is a validated tool to use in pregnancy and consists of 20 items with each item being scored between 0 and 3 on a four-point Likert scale, from respectively ‘rarely or none’ to ‘almost all the time’. Total score on the CES-D questionnaire can range from 0 to 60, with a score of ≥ 16 being suggestive for clinical depression [21]. 

Questionnaire V on anxiety: The validated six-item short-form of the Spielberger State-Trait Anxiety Inventory (STAI-6) questionnaire was administered at each point in time to measure state anxiety level. The six items are scored from ‘very much’ to ‘not at all’ with a four-point Likert scale and a total score ranging from 6 to 24, with a higher score referring to a greater level of anxiety [22,23]. 

Questionnaire VI on treatment satisfaction: The Diabetes Treatment Satisfaction Questionnaire—status version (DTSQs) is a validated tool for measuring satisfaction with diabetes treatment regimens and was administered after the follow-up session. This is an eight-item questionnaire in which each item is scored on a seven-point Likert scale from 0 to 6 [24]. The DTSQs scale score is calculated by summing the six satisfaction item scores. Total scores can range between 0 and 36, with higher scores indicating better treatment satisfaction. Item 2 and 3 are related to perceived frequency of hyperglycemia and hypoglycemia and are analyzed separately. They are reported on a seven-point Likert scale, with lower scores indicating fewer episodes of hyperglycemia or hypoglycemia [25].

#### 2.3.2. Data from the Electronic Medical Records

Data from the participant’s electronic medical record (EMR) were collected during pregnancy, at delivery and at three months postpartum. Maternal characteristics recorded were age, ethnicity, height, body weight, body mass index (BMI) at first prenatal visit and at delivery, overweight (BMI ≥ 25 kg/m^2^), obesity (BMI ≥ 30 kg/m^2^), gestational weight gain (difference in weight between delivery and first prenatal visit), parity, family history of diabetes, smoking, alcohol intake during pregnancy and history of GDM. Excessive gestational weight gain was defined according to the most recent Institute of Medicine (IOM) guidelines [26].

Data recorded about the diagnosis and treatment of GDM were timing and result of the GCT and OGTT, gestational age at the diagnosis of GDM, HbA1c at the time of the OGTT during pregnancy, time between diagnosis and start of the treatment, whether women received treatment with corticoids during pregnancy, need of insulin, type of insulin and gestational age at the start of insulin, timing and result of the postpartum OGTT.

The following maternal pregnancy outcomes were recorded: gestational hypertension (blood pressure ≥ 140/90 mmHg), preeclampsia (hypertension with proteinuria or in combination with reduced fetal growth or the ‘Hemolysis Elevated Liver enzymes and Low Platelets’ (HELPP)-syndrome), preterm delivery (<37 weeks of gestation) and cesarean section (planned and emergency sections combined). Neonatal pregnancy outcomes recorded were: gender, birth weight, macrosomia (birth weight > 4 kg), large-for-gestational age infants (LGA, birth weight > 90 percentile adjusted for sex and parity according to the Flemish birth charts), small-for-gestational age infants (SGA, birth weight < 10 percentile adjusted for sex and parity according to the Flemish birth charts), shoulder dystocia, Apgar score at five minutes and admission at the neonatal intensive care unit (NICU) [27].

### 2.4. Statistical Analyses

Statistical analyses were performed using SPSS software for Windows (IBM SPSS Statistics version 25.0, Armonk, NY, USA). Continuous data were expressed as mean and standard deviation (SD) if normally distributed, otherwise, variables were displayed as median and interquartile range (IQR). Categorical data were presented as frequencies and percentages. To compare variables between two groups, independent sample t-tests were used for normally distributed continuous variables, Mann-Whitney U-tests for non-normal variables and Chi-square tests for categorical variables. To evaluate the effect of the education, the Wilcoxon Signed Rank test was used for non-normal variables and McNemar test for categorical variables. Multivariable models were used to correct for significant differences in general patient characteristics between both groups. Linear regression was used for continuous outcomes and logistic regression for binary outcomes. A *p*-value of <0.05 (two-tailed) was considered significant.

## 3. Results

A total of 200 pregnant women with a recent diagnosis of GDM were enrolled. The prevalence of GDM over this period was 6.6% (*n* = 392). Women with GDM who did not participate in the study despite meeting the inclusion criteria (*n* = 145), were mostly women who received the initial education during hospitalization, were unable to participate due to practical reasons such as arriving too late for the education session, or women who declined to participate. Of all participants, 100 attended the initial education session in group and 100 received the initial education session individually. As this was an observational study, the equal division of participants over the two groups occurred by chance. The average number of women seen in each group education session was three. Results did not differ when excluding non-Dutch speaking women. The supplementary (Tables S1–S8), gives an overview of the general characteristics and comparisons between the individual and group education groups excluding non-Dutch-speaking women from the analyzes.

### 3.1. General Characteristics 

The mean age of the participants was 32.3 ± 5.0 years, 11.2% (*n* = 13 of 116 women with more than one pregnancy) had a previous history of GDM, 23.7% (*n* = 47) had an ethnic minority (EM) background. The most frequent EM background was Asian in 10.6% (*n* = 21), Black-African in 4.0% (*n* = 8), Northern-African in 4.0% (*n* = 8), Middle-Eastern in 2.0% (*n* = 4) and Turkish in 1.5% (*n* = 3). Women receiving individual education were more often from an EM background than women in group education (32.0% (*n* = 32) versus 15.3% (*n* = 15), *p* = 0.01). There were no other significant differences in characteristics between women in each setting (Table 2). Of all women with GDM, 85.0% (*n* = 170) attended the postpartum OGTT of which 40.4% (*n* = 67) had glucose intolerance (Table 2).

### 3.2. Pregnancy Outcomes 

Of all woman, 21.9% (*n* = 41) had excessive gestational weight gain, 8.2% (*n* = 16) had preeclampsia and 30.3% (*n* = 59) had a cesarean section. Of all babies, 9.3% (*n* = 18) was LGA, 9.9% (*n* = 19) SGA and 5.2% (*n* = 10) had macrosomia. There were no significant differences in pregnancy outcomes between the different education groups (Table 3).

### 3.3. Knowledge about GDM

Of all participants, 159 (79.5%) completed the knowledge questionnaire prior to the initial session and after the follow-up session. Women generally had good knowledge about GDM before the initial education session, which improved significantly for almost all items after the follow-up session (Table 4).

Women showed an overall good knowledge of most topics after the initial education session, with almost no significant differences between the group and individual education groups (Table 5).

### 3.4. Satisfaction with the Education and Treatment

#### 3.4.1. Satisfaction with the Education Sessions 

Patients were overall satisfied with the content and duration of both the initial and follow-up education sessions (Table 6). The majority of all women were satisfied with the explanation that was given on the subject of pathophysiology, risks, treatment, and follow-up of GDM. There were no significant differences in satisfaction rates after the initial education session between women receiving group education and those receiving individual education (Table 6). 

Of all women who completed the additional questions about group education (*n* = 98), 91.8% (*n* = 90) were pleased with the size of the group. A large majority of 76.5% (*n* = 75) indicated that group education fulfilled their expectations, although 22% (*n* = 22) indicated that they would prefer supplementary individual education after group education. Only four women (4.1%) preferred individual education alone. The most frequently reported advantages of group education were ‘learning from the questions of others’ (74.5%, *n* = 73) and ‘learning from the experience of others’ (50.0%, *n* = 49), followed by ‘feeling supported by the group’ (26.5%, *n* = 26) and ‘helping you to stick to the advice’ (14.3%, *n* = 14). However, 10 women (10.2%) reported no advantages of group education and four women (4.1%) indicated that they felt inhibited by the group.

#### 3.4.2. Satisfaction with the Treatment Regimen

Of all responders (*n* = 152), a large majority agreed that is was possible to follow the advice about diet, physical activity, weight gain, and glycemic measurements. Almost all participants agreed or strongly agreed with the statement that they were confident in the given advice (96.7%, *n* = 147). However, 23.0% (*n* = 35) perceived the advice to be too strict and indicated that they felt starved. There were no significant differences in agreement with the feasibility of the advice between women who received group education and those who received individual education (Table 7).

Mean total score of the DTSQs was 27.3 (± 0.5) and this was not significantly different for women in group or individual education (27.1 ± 5.4 versus 27.5 ± 5.6, *p* = 0.692). Mean total score for the two items on perceived hyper- or hypoglycemia was 3.1 (± 2.1) and this was not significantly different between both education groups (2.9 ± 1.8 versus 3.3 ± 2.4, *p* = 0.240).

### 3.5. Emotional Status

Of all responders (*n* = 148), 25.0% (*n* = 37) had a total score ≥ 16 at the CES-D questionnaire prior to the initial education session and were therefore considered at risk for clinical depression. This percentage declined to 18.9% (*n* = 28) after the follow-up session (*p* = 0.137). The median total score on the STAI-6 questionnaire decreased significantly from 12 (10–14) at the start of the initial education session to 11 (8–13) at the end of the follow-up session (*p* < 0.0001).

No significant differences in clinical depression rates (25.6% versus 24.2%, *p* = 0.967) or median anxiety scores (11.0 versus 10.5, *p* = 0.294) were observed between both education groups after the initial education session.

### 3.6. Comments on the Education Sessions

In general, most women indicated that the explanation was sufficient and felt that all of their questions had been addressed during the education sessions. However, topics such as postpartum follow-up and future risks should have been addressed in more detail according to a few women. Another recurring comment was the demand for specific recipes and nutritional instructions. Women often indicated that they were well aware of what they should not eat, but struggled with deciding what they were allowed to eat instead.

## 4. Discussion

Due to the worldwide obesity epidemic and the adoption of the 2013 WHO diagnostic criteria, the prevalence of GDM will continue to increase. This contributes to a number of practical challenges in the management of GDM, such as an increased workload for health care providers and a growing demand for additional resources [11,28]. In order to cope with this increasing burden, it may be useful to reconsider the way in which health care services are currently provided for women with newly diagnosed GDM. Group education is a commonly used approach in the treatment of diabetes, but little is known about the effectiveness of group education in women with GDM in particular. However, gaining sufficient insight into the perceptions of GDM patients with regard to their treatment is crucial to better organize educational programs that are adapted to their needs.

Improved knowledge about GDM in newly diagnosed women might result in better adoption of a healthy lifestyle, better treatment adherence and better self-management. A Malaysian study among 175 women with GDM demonstrated that better knowledge about GDM is related to better glycemic control [29]. Our study showed that a multidisciplinary education program for the management of GDM can effectively improve the knowledge on GDM with almost no difference whether women received group education or individual education. This finding is consistent with a recent Irish study investigating the effectiveness of group education on knowledge of women with newly diagnosed GDM [19]. However, no comparison was made with individual education in the Irish study. A Canadian study further investigated the impact of small-group versus individual nutritional counseling on knowledge improvement of women with GDM and showed that women with GDM can be effectively and cost-efficiently counselled on nutrition in small-group settings. [30].

Patient satisfaction is an important consideration in the organization of medical care, since improved satisfaction rates appear to be associated with a more effective engagement in health care programs [31]. In this prospective study, participants were overall satisfied with the content and duration of both the initial and follow-up education sessions, independent of whether they received group or individual education. The majority agreed with the clarity and relevance of the explanation given about the different aspects of GDM. Furthermore, almost all participants were confident in the given advice. However, a considerable group of women (23.0%) perceived the advice to be too strict and indicated that they felt starved. The same observations were made in the Italian DAWN (Diabetes Attitudes, Wishes, and Needs) Pregnancy Study, which was promoted by the International Diabetes Federation to evaluate the quality of life, wishes and needs in women diagnosed with GDM [32]. In this study, women indicated that they experienced difficulties in following the treatment regimen and that the dietary advice was one of their biggest concerns. This study also showed that the issue of eating habits among immigrant women with GDM is often more difficult compared to indigenous women. To address these concerns, we have developed leaflets in cooperation with specialized diabetes dietitians on specific dietary guidelines and with adapted recipes for Flemish, Asian, and North-African cuisine. 

The results of the DTSQs revealed that the participants were generally very satisfied with their treatment, whether they received group or individual education. A Malaysian study in women with GDM showed that higher treatment satisfaction is associated with better glycemic control [25]. Other studies in the field of diabetes research have demonstrated that treatment satisfaction can have a significant impact on clinical outcomes as well as on treatment adherence [33,34]. Determining patient treatment satisfaction levels could therefore be a useful tool in improving healthcare delivery in patients with GDM. 

More than 90% of all group participants were satisfied with the group size and almost 80% indicated that group education met their expectations. Only a small minority reported that they felt inhibited by the group and preferred individual education. This is in contrast to the findings of a study from New Zealand, establishing that only a minority of the people surveyed would consider participating in a group session, fearing that less attention would be paid to the individual needs of each patient [35]. In our study, the most important advantages reported with group education were learning from others’ questions and experiences. This is in line with another observational study on GDM, showing that the beneficial effects of their prenatal group care program were associated with factors as learning from the experience of peers, a greater connection with health care providers, and a motivating group dynamic [18].

Depression is a common condition in women with GDM, with studies reporting depression rates between 15% and 20% in this population [36,37]. In our cohort, 25% of all women were considered at risk for clinical depression prior to the initial education session, which declined to 18.9% after the follow-up session, although this was not statistically significant. Depression can lead to poor management of GDM, thus increasing the risk of adverse pregnancy outcomes such as macrosomia and neonatal hypoglycemia [38]. Health care providers should therefore be aware of the risk of antenatal depression when treating women with GDM and should regularly screen for depression in order to timely provide appropriate care. GDM can also cause maternal anxiety and stress related to the perception of a high-risk pregnancy, fear of maternal and neonatal complications and the feeling of losing control during the process of dietary management [39]. However, feelings of anxiety tend to decrease throughout pregnancy, which could be related to the understanding that GDM is a self-limiting condition and which might suggest that education and reassurance by health care providers is successful in dissipating anxiety in women diagnosed with GDM [40]. This is in line with the findings in our study, as feelings of anxiety were apparent prior to the initial education but declined significantly after education was given. To our knowledge, no studies have been conducted to compare feelings of depression and anxiety between women with GDM in group versus individual education. We show that there were no significant differences in terms of feelings of depression and anxiety between patients receiving group education and those receiving individual education.

We present the first prospective study on the impact of group education on patient’s knowledge about GDM, their satisfaction with the education and treatment, and emotional status. Our study demonstrates that group education is at least as good an alternative to individual education with regard to these outcomes. Moreover, our results did not differ when non-Dutch speaking women were excluded from the analyzes.

Our study has several strengths. We provide prospective data of a large cohort of women with GDM, allowing the evaluation of the impact of group education compared to individual education. In addition, we used several validated questionnaires to evaluate treatment satisfaction and emotional status of women with GDM. When validated questionnaires were not available, we used self-designed questionnaires based on our experience and previous research in women with GDM. We included French and English speaking women in our cohort, ensuring that our results are representative of a multi-ethnic population. However, we could not evaluate group education for non-Dutch-speaking, since group education was only offered to Dutch-speaking women in line with routine care in our hospital. 

A limitation of our study is the observational design. Women following individual education were more often from an EM background than women in group education, but we corrected for this difference through multiple regression analysis. In addition, some participation bias is likely since not all eligible patients participated in the study and we have no data available on their characteristics. 

## 5. Conclusions

The results of this study show that women with newly diagnosed GDM are overall satisfied with their education and treatment, and have a better understanding of their condition after education, independent of the education setting. We show that group education is a valuable alternative to better organize education in view of the increasing GDM prevalence and is perceived as an added value by GDM patients.

## Figures and Tables

**Table 1:** Overview of the different self-administered questionnaires at the different visits.

	Prior to the Initial Session	after the Initial Session	after the Follow-Up Session
I: questionnaire on sociodemographic characteristics	x		
II: questionnaire on knowledge about GDM	x	x	x
IIIa: questionnaire on satisfaction with the initial session		x	
IIIb: questionnaire on satisfaction with the follow-up session			x
IV: CES-D questionnaire on depression	x	x	x
V: STAI-6 questionnaire on anxiety	x	x	x
VI: DTSQs questionnaire on treatment satisfaction			x

GDM: gestational diabetes mellitus; CES-D: Center for Epidemiologic Studies Depression; STAI: Spielberger State-Trait Anxiety Inventory; DTSQs: Diabetes Treatment Satisfaction Questionnaire—status version.

**Table 2:** Baseline characteristics.

	General Cohort *n* = 200	Group Education *n* = 100	Individual Education *n* = 100	*p*-Value
age (mean ± SD)	32.3 ± 5.0	32.1 ± 5.3	23.6 ± 4.7	0.455
BMI at first prenatal visit (mean ± SD)	26.7 ± 5.4	26.5 ± 5.4	26.8 ± 5.4	0.695
overweight at first prenatal visit	30.6 (60)	29.3 (29)	32.0 (31)	0.917
obese at first prenatal visit % (*n*)	27.0 (53)	27.3 (27)	26.8 (26)	0.917
EM background % (*n*)	23.7 (47)	15.3 (15)	32.0 (32)	**0.010**
primigravida % (*n*)	39.9 (79)	44.0 (44)	35.7 (35)	0.410
first degree relative with T2DM % (*n*)	20.2 (40)	17.0 (17)	23.5 (23)	0.526
history of GDM (*n* = 116) % (*n*)	11.2 (13)	13.0 (7)	9.7 (6)	0.913
high secondary diploma % (*n*)	84.8 (168)	84.8 (84)	84.8 (84)	0.166
higher degree diploma % (*n*)	71.5 (138)	69.8 (67)	73.2 (71)	0.716
paid job % (*n*)	81.0 (158)	83.7 (82)	78.4 (76)	0.409
single % (*n*)	7.1 (14)	7.1 (7)	7.1 (7)	1.000
week at OGTT (median (IQR))	26 (24–28)	26 (25–27)	26 (24–28)	0.617
Hba1c % at OGTT (mean ± SD)	5.2 ± 0.4	5.2 ± 0.4	5.2 ± 0.4	0.768
insulin treatment % (*n*)	17.2 (33)	16.3 (16)	18.1 (17)	0.895
present at postpartum OGTT % (*n*)	85.0 (170)	88.0 (88)	82.0 (82)	0.990 *
weeks after delivery (median (IQR))	14 (10–18)	15 (12–18)	14 (9–19)	0.398 *
abnormal postpartum OGTT % (*n*)	40.4 (67)	35.6 (31)	45.6 (36)	0.284 *

GDM: gestational diabetes mellitus; BMI = body mass index; OGTT: oral glucose tolerance test; EM: ethnic minority; T2DM: type 2 diabetes mellitus; Statistically significant *p*-values are in bold; *p-values based on multiple linear and logistic regression adjusting for difference in EM background.

**Table 3:** Pregnancy outcomes of women in group versus individual education.

	General Cohort *n* = 200	Group Education *n* = 100	Individual Education *n* = 100	*p*-Value
**Maternal Outcomes**				
total weeks of gestation (median)	39 (38–40)	39 (37–41)	39 (38–40)	0.803 *
excessive weight gain % (*n*)	21.9 (42)	18.2 (18)	25.8 (24)	0.847 *
gestational hypertension % (*n*)	13.8 (27)	16.0 (16)	11.6 (11)	0.401 *
preeclampsia % (*n*)	8.2 (16)	12.0 (12)	4.2 (4)	0.075 *
preterm delivery % (*n*)	9.7 (19)	12.0 (12)	7.4 (7)	0.407 *
cesarean section % (*n*)	30.3 (59)	31.0 (31)	29.5 (28)	0.415 *
**Neonatal Outcomes**				
macrosomia % (*n*)	5.2 (10)	5.0 (5)	5.3 (5)	1.000
LGA % (*n*)	9.3 (18)	6.1 (6)	12.8 (12)	0.069 *
SGA % (*n*)	9.9 (19)	9.1 (9)	10.8 (10)	0.764 *
shoulder dystocia % (*n*)	2.1 (4)	2.0 (2)	2.1 (2)	1.000
NICU transfer % (*n*)	6.2 (12)	7.0 (7)	5.4 (5)	0.774 *
Apgar score < 7 after 5 minutes % (*n*)	3.2 (6)	1.0 (1)	5.6 (5)	0.104

LGA: large for gestational age; SGA: small for gestational age; NICU: neonatal intensive care unit; Statistically significant *p*-values are in bold; * *p*-values based on multiple linear and logistic regression adjusting for difference in EM background.

**Table 4:** Comparison of the correct responses on the knowledge questionnaire prior to the initial education session and after the follow-up session.

	Prior to the Initial Session *n* = 159 % (*n*)	after the Follow-Up Session *n* = 159 % (*n*)	*p*-Value
**When is GDM diagnosed?**			
24–28 weeks	95.6 (152)	98.7 (157)	0.180
**How is GDM diagnosed?**			
Based on a fasting blood collection in combination with drinking a sugar solution	82.4 (131)	87.4 (139)	0.201
**It’s more likely to develop gestational diabetes if you:**			
Are overweight before pregnancy	78.0 (124)	87.4 (139)	**0.007**
Gain too much weight during the pregnancy	35.2 (56)	53.5 (85)	**<0.0001**
Have had GDM during a previous pregnancy	71.1 (113)	79.9 (127)	**0.011**
Have a first degree relative with diabetes	75.5 (120)	80.5 (128)	0.152
Your age is > 30 years	49.7 (79)	63.5 (101)	**0.002**
**What are the consequences for the baby if the treatment of GDM is insufficient?**			
Too high birth weight of the baby	85.5 (136)	97.5 155)	**<0.0001**
Increased risk of diabetes for the baby later on	60.4 (96)	74.2 (118)	**0.006**
Increased risk of overweight for the baby later on	50.9 (81)	69.8 (111)	**<0.0001**
**What are the risks for you if the treatment of GDM is insufficient?**			
An increased risk for a difficult delivery	77.7 (122)	91.7 (144)	**<0.0001**
An increased risk for preeclampsia	22.2 (35)	75.9 (120)	**<0.0001**
An increased risk for a cesarean section	66.5 (105)	89.9 (142)	**<0.0001**
**How GDM is initially treated after diagnosis?**			
Dietary change and increasing physical activity	59.1 (94)	79.2 (126)	**<0.0001**
Insulin is only started if dietary change and physical activity is insufficient	53.5 (85)	63.5 (101)	**0.049**
**Which food products do you have to restrict if you have GDM?**			
Pie	88.1 (140)	95.6 (152)	**0.008**
Fruits	19.5 (31)	54.1 (86)	**<0.0001**
Sugared soda	94.3 (150)	98.1 (156)	**0.031**
Fruit juice	74.8 (119)	93.1 (148)	**<0.0001**
**Which fasting blood sugar level is normal in the morning?**			
< 95 mg/dl	39.0 (62)	98.1 (156)	**<0.0001**
**Which blood sugar level is normal 2 hours after eating?**			
< 120 mg/dl	25.8 41)	96.2 (153)	**<0.0001**
**How can best be checked if your blood sugar levels are sufficiently under control?**			
Based on a finger prick with a glucometer	79.0 (124)	98.7 (155)	**<0.0001**
**What do you think about the treatment with insulin for GDM?**			
This can lower the risk of an overweight baby	45.9 (72)	73.2 (115)	**<0.0001**
**What do you think about breastfeeding after a pregnancy with GDM?**			
This is good for the general health of the baby	58.5 (93)	89.9 (143)	**<0.0001**
This can lower the risk of diabetes and overweight in the baby later on	25.2 (40)	37.7 (60)	**0.002**
**What do you think that happens with your GDM after your delivery?**			
GDM disappears completely but I have a strongly increased risk of 50% to develop T2DM within 10 years	39.0 (62)	83.0 (132)	**<0.0001**

GDM: gestational diabetes mellitus; T2DM: type 2 diabetes mellitus; Statistically significant *p*-values are in bold.

**Table 5:** Comparison of correct responses on the knowledge questionnaire between group and individual education after the first education session.

	Group Education *n* = 98 % (*n*)	Individual Education *n* = 99 % (*n*)	*p*-Value
**When is GDM diagnosed?**			
24–28 weeks	99.0 (97)	98.0 (97)	1.000
**How is GDM diagnosed?**			
Based on a fasting blood collection in combination with drinking a sugar solution	87.8 (86)	88.9 (88)	0.979
**It’s more likely to develop gestational diabetes if you:**			
Are overweight before pregnancy	91.8 (90)	77.8 (77)	**0.011**
Gain too much weight during the pregnancy	38.8 (38)	40.4 (40)	0.930
Have had GDM during a previous pregnancy	77.6 (76)	65.7 (65)	0.091
Have a first degree relative with diabetes	89.8 (88)	75.8 (75)	**0.016**
Your age is > 30 years	62.2 (61)	52.5 (52)	0.217
**What are the consequences for the baby if the treatment of GDM is insufficient?**			
Too high birth weight of the baby	95.9 (94)	94.9 (94)	1.000
Increased risk of diabetes for the baby later on	80.6 (79)	75.8 (75)	0.514
Increased risk of overweight for the baby later on	73.5 (72)	70.7 (70)	0.785
**What are the risks for you if the treatment of GDM is insufficient?**			
An increased risk for a difficult delivery	91.8 (90)	90.9 (90)	1.000
An increased risk for preeclampsia	92.9 (91)	93.8 (83)	0.080
An increased risk for a cesarean section	90.8 (89)	90.9 (90)	1.000
**How is GDM initially treated after diagnosis?**			
Dietary change and increasing physical activity	81.6 (80)	79.8 (79)	0.884
Insulin is only started if dietary change and physical activity is insufficient	74.5 (73)	60.6 (60)	0.054
**Which food products do you have to restrict if you have GDM?**			
Pie	96.9 (96)	91.9 (91)	0.221
Fruits	63.6 (62)	51.5 (51)	0.128
Sugared soda	95.9 (94)	94.9 (94)	1.000
Fruit juice	98.0 (96)	94.9 (94)	0.445
**Which fasting blood sugar level is normal in the morning?**			
< 95 mg/dl	98.0 (96)	92.9 (92)	0.170
**Which blood sugar level is normal 2 hours after eating?**			
< 120 mg/dl	96.9 (96)	91.9 (91)	0.221
**How can best be checked if your blood sugar levels are sufficiently under control?**			
Based on a finger prick with a glucometer	94.9 (93)	94.9 (94)	1.000
**What do you think about the treatment with insulin for GDM?**			
This can lower the risk of an overweight baby	77.6 (76)	71.7 (71)	0.437
**What do you think about breastfeeding after a pregnancy with GDM?**			
This is good for the general health of the baby	85.7 (84)	93.9 (92)	0.099
This can lower the risk of diabetes and overweight in the baby later on	32.7 (32)	34.7 (34)	0.880
**What do you think that happens with your GDM after your delivery?**			
GDM disappears completely but I have a strongly increased risk of 50% to develop T2DM within 10 years	85.7 (84)	86.9 (86)	0.977

GDM: gestational diabetes mellitus; T2DM: type 2 diabetes mellitus; Statistically significant *p*-values are in bold.

**Table 6:** Satisfaction rates with the given explanation on 12 items after the initial education session for women in group education compared to women in individual education.

	Strongly Disagree		Disagree		Neutral		Agree		Strongly Agree		I Don’t Know		*p*-Value
	G % (*n*)	I % (*n*)	G % (*n*)	I % (*n*)	G % (*n*)	I % (*n*)	G % (*n*)	I % (*n*)	G % (*n*)	I % (*n*)	G % (*n*)	I % (*n*)	
**Q1: What is GDM**	0.0 (0)	1.0 (1)	0.0 (0)	0.0 (0)	2.0 (2)	0.0 (0)	21.4 (21)	18.4 (18)	75.5 (74)	80.6 (79)	1.0 (1)	0.0 (0)	0.568
**Q2: Importance of treatment**	0.0 (0)	1.0 (1)	0.0 (0)	0.0 (0)	3.1 (3)	0.0 (0)	16.3 (16)	14.3 (14)	80.6 (79)	84.7 (83)	0.0 (0)	0.0 (0)	0.426
**Q3: Risks for myself**	0.0 (0)	1.0 (1)	1.0 (1)	0.0 (0)	4.1 (4)	1.0 (1)	20.4 (20)	15.5 (15)	73.5 (72)	82.5 (80)	1.0 (1)	0.0 (0)	0.212
**Q4: Risks for my baby**	0.0 (0)	1.0 (1)	0.0 (0)	0.0 (0)	2.0 (2)	0.0 (0)	21.4 (21)	19.4 (19)	75.5 (74)	79.6 (78)	1.0 (1)	0.0 (0)	0.688
**Q5: Treatment with diet**	0.0 (0)	1.0 (1)	0.0 (0)	0.0 (0)	4.1 (4)	2.0 (2)	32.7 (32)	23.5 (23)	63.3 (62)	73.5 (72)	0.0 (0)	0.0 (0)	0.131
**Q6: Treatment with physical activity**	0.0 (0)	1.0 (1)	1.0 (1)	0.0 (0)	3.1 (3)	2.0 (2)	28.6 (28)	27.6 (27)	66.3 (65)	69.4 (68)	1.0 (1)	0.0 (0)	0.817
**Q7: Weight gain**	0.0 (0)	2.1 (2)	2.0 (2)	0.0 (0)	12.2 (12)	3.1 (3)	29.6 (29)	37.1 (36)	55.1 (54)	57.7 (56)	1.0 (1)	0.0 (0)	0.534
**Q8: Measuring blood sugar levels**	0.0 (0)	1.0 (1)	1.0 (1)	0.0 (0)	1.0 (1)	0.0 (0)	17.3 (17)	18.8 (18)	79.6 (78)	80.2 (77)	1.0 (1)	0.0 (0)	0.857
**Q9: Treatment with insulin**	0.0 (0)	1.0 (1)	3.1 (3)	4.2 (4)	23.7 (23)	11.5 (11)	34.0 (33)	35.4 (34)	37.1 (36)	44.8 (43)	2.1 (2)	3.1 (3)	0.129
**Q10: Follow-up after delivery**	0.0 (0)	1.0 (1)	1.0 (1)	0.0 (0)	2.0 (2)	2.0 (2)	31.6 (31)	28.6 (28)	64.3 (63)	68.4 (67)	1.0 (1)	0.0 (0)	0.741
**Q11: Risk of diabetes after delivery**	0.0 (0)	1.0 (1)	1.0 (1)	0.0 (0)	4.1 (4)	0.0 (0)	25.5 (25)	26.5 (26)	68.4 (67)	71.4 (70)	1.0 (1)	1.0 (1)	0.539
**Q12: Breastfeeding**	0.0 (0)	2.1 (2)	3.1 (3)	1.0 (1)	3.1 (3)	2.1 (2)	27.6 (27)	23.7 (23)	66.3 (65)	71.1 (69)	0.0 (0)	0.0 (0)	0.481

Q = question; G: group education; I: individual education; GDM: gestational diabetes mellitus.

**Table 7:** Agreement with the feasibility of the advice for the management of GDM.

	Strongly Disagree		Disagree		Neutral		Agree		Strongly Agree		I Don’t Know		*p*-Value
	G % (*n*)	I % (*n*)	G % (*n*)	I % (*n*)	G % (*n*)	I % (*n*)	G % (*n*)	I % (*n*)	G % (*n*)	I % (*n*)	G % (*n*)	I % (*n*)	
**Advice about diet**	0.0 (0)	0.0 (0)	0.0 (0)	1.2 (1)	8.5 (6)	4.9 (4)	36.6 (26)	42.0 (34)	54.9 (39)	51.9 (42)	0.0 (0)	0.0 (0)	0.821
**Advice about physical activity**	0.0 (0)	0.0 (0)	5.6 (4)	6.3 (5)	11.1 (8)	8.9 (7)	43.1 (31)	41.8 (33)	40.3 (29)	43.0 (34)	0.0 (0)	0.0 (0)	0.733
Advice about treatment with insulin *	20.0 (1)	0.0 (0)	0.0 (0)	0.0 (0)	0.0 (0)	12.5 (1)	60.0 (3)	37.5 (3)	0.0 (0)	12.5 (1)	20.0 (1)	37.5 (3)	0.435
**Advice about weight gain**	0.0 (0)	0.0 (0)	1.4 (1)	0.0 (0)	5.8 (4)	11.3 (9)	43.5 (30)	40.0 (32)	49.3 (34)	46.3 (37)	0.0 (0)	2.5 (2)	0.896
**Advice about SMBG**	0.0 (0)	0.0 (0)	0.0 (0)	0.0 (0)	1.4 (1)	1.3 (1)	36.1 (26)	31.3 (25)	62.5 (45)	67.5 (54)	0.0 (0)	0.0 (0)	0.523
**The advice was too strict and I felt starved**	22.5 (16)	13.6 (11)	38.0 (27)	43.2 (35)	18.3 (13)	16.0 (13)	14.1 (10)	11.1 (9)	5.6 (4)	14.8 (12)	1.4 (1)	1.2 (1)	0.212
**I am confident in the given advice**	1.4 (1)	0.0 (0)	0.0 (0)	0.0 (0)	4.2 (3)	1.2 (1)	35.2 (25)	42.0 (34)	59.2 (42)	56.8 (46)	0.0 (0)	0.0 (0)	0.959

* Only those who were treated with insulin and filled in the questionnaire (*n* = 13); G: group education; I: individual education; GDM: gestational diabetes mellitus; SMBG: self-monitoring of blood glucose.

## Supplementary Materials

The following are available online at https://www.mdpi.com/2077-0383/9/2/509/s1, Table S1: Baseline characteristics, Table S2: Pregnancy outcomes of women in group versus individual education, Table S3: Comparison of the correct responses on the knowledge questionnaire prior to the initial education session and after the follow-up session, Table S4: Comparison of correct responses on the knowledge questionnaire between group and individual education after the first education session, Table S5: Satisfaction rates with the given explanation on 12 items after the initial education session for women in group education compared to women in individual education, Table S6: Agreement with the feasibility of the advice for the management of GDM for women in group education compared to women in individual education, Table S7: Emotional status before the initial education session versus after the follow-up session for the complete Dutch cohort, Table S8: Emotional status in group versus individual education at the end of the initial education session.
