# Supplementary material for: Multidisciplinary Group Education for Gestational Diabetes Mellitus: A Prospective Observational Cohort Study
Source: J Clin Med. 2020 Feb 13;9(2):509. doi: 10.3390/jcm9020509 (PMC7073549; doi:10.3390/jcm9020509)
Supplement: Supplementary file 1 [file jcm-09-00509-s001.pdf]

Journal of
Clinical Medicine

MDPI

Supplementary: Overview of the general characteristics and comparisons between the individual and group education groups excluding non-Dutch-speaking women from the analyzes.

Table S1. Baseline characteristics.

	General Cohort *n* = 172	Group Education *n* = 98	Individual Education *n* = 74	*p*-Value
age (mean ± SD)	32.3±5.1	32.1±5.3	32.6±4.9	0.522
BMI at first prenatal visit (mean ± SD)	26.8±5.4	26.5±5.4	27.1±5.4	0.968
overweight at first prenatal visit %(n)	30.1 (52)	29.3 (29)	31.1 (23)	0.853
obese at first prenatal visit %(n)	28.3 (49)	27.3 (27)	29.7 (22)	0.853
EM background %(n)	16.3 (28)	15.3 (15)	17.6 (13)	0.850
primigravida %(n)	40.2 (70)	44.0 (44)	35.1 (26)	0.223
first degree relative with T2DM %(n)	19.5 (34)	17.0 (17)	23.0 (17)	0.577
history of GDM (n = 102) %(n)	10.8 (11)	13.0 (7)	8.3 (4)	0.823
high secondary diploma %(n)	84.3 (145)	84.8 (84)	83.6 (61)	0.253
higher degree diploma %(n)	68.9 (115)	69.8 (67)	67.6 (48)	0.866
paid job %(n)	84.7 (144)	83.7 (82)	86.1 (62)	0.825
single %(n)	7.6 (13)	7.1 (7)	8.2 (6)	1.000
week at OGTT (median (IQR))	26 (24-28)	26 (25-27)	26 (24-28)	0.743
Hba1c % at OGTT (mean ± SD)	5.2 ± 0.4	5.2 0.4	5.2 ± 0.4	0.774
insulin treatment %(n)	15.3 (26)	16.3 (16)	13.9 (10)	0.825
present at postpartum OGTT %(n)	89.7 (56)	88.0 (88)	91/9 (68)	0.246
weeks after delivery (median (IQR))	14 (9-19)	15 (12-18)	14 (8-20)	0.804
abnormal postpartum OGTT %(n)	38.2 (58)	35.6 (31)	41.5 (27)	0.567

GDM: gestational diabetes mellitus; BMI = body mass index; OGTT: oral glucose tolerance test; EM: ethnic minority; T2DM: type 2 diabetes mellitus; Statistically significant p-values are in bold.

Table S2. Pregnancy outcomes of women in group versus individual education.

	general cohort n = 172	group education n = 98	individual education n = 74	p-value
**Maternal Outcomes**				
total weeks of gestation (median)	39 (37-41)	39 (37-41)	39 (38-40)	0.804
gestational hypertension %(n)	14.5 (25)	16.0 (16)	12.3 (9)	0.646
preeclampsia %(n)	9.2 (16)	12.0 (12)	5.5 (4)	0.232
preterm delivery %(n)	9.2 (16)	12.0 (12)	5.5 (4)	0.232
cesarean section %(n)	29.5 (51)	31.0 (31)	27.4 (20)	0.730
**Neonatal Outcomes**				
macrosomia %(n)	5.2 (9)	5,0 (5)	5.6 (4)	1.000
LGA %(n)	8.2 (14)	6.1 (6)	11.1 (8)	0.364
SGA %(n)	10.0 (17)	9.1 (9)	11.3 (8)	0.836
shoulder dystocia %(n)	1.7 (3)	2.0 (2)	1.4 (1)	1.000
NICU transfer %(n)	6.4 (11)	7.0 (7)	5.6 (4)	1.000
Apgar score < 7 after 5 minutes %(n)	1.8 (3)	1.0 (1)	2.9 (2)	0.569

LGA: large for gestational age; SGA: small for gestational age; NICU: neonatal intensive care unit; Statistically significant p-values are in bold.

Table S3. Comparison of the correct responses on the knowledge questionnaire prior to the initial education session and after the follow-up session.

	prior to the initial session n = 138 % (n)	after the follow-up session n = 138 % (n)	p-value
**When is GDM diagnosed?**			
24 - 28 weeks	97.1 (134)	100.0 (138)	0.125
**How is GDM diagnosed?**			
Based on a fasting blood collection in combination with drinking a sugar solution	84.1 (116)	87.0 (120)	0.541
**It's more likely to develop gestational diabetes if you:**			
Are overweight before pregnancy	81.9 (113)	88.4 (122)	0.064
Gain too much weight during the pregnancy	34.8 (48)	50.7 (70)	**0,001**
Have had GDM during a previous pregnancy	74.6 (103)	80.4 (11)	0,115
Have a first degree relative with diabetes	77.5 (107)	84.1 (116)	**0,001**
Your age is > 30 years	50.0 (69)	67.4 (93)	**<0,0001**
**What are the consequences for the baby if the treatment of GDM is insufficient?**			
Too high birth weight of the baby	87.7 (121)	97.8 (135)	**0.0005**
Increased risk of diabetes for the baby later on	59.4 (82)	75.4 (104)	**0.004**
Increased risk of overweight for the baby later on	51.4 (71)	71.7 (99)	**<0.0001**
**What are the risks for you if the treatment of GDM is insufficient?**			
An increased risk for a difficult delivery	78.7 (107)	90.4 (123)	**0.003**
An increased risk for preeclampsia	21.2 (29)	78.8 (108)	**<0.0001**
An increased risk for a cesarean section	69.3 (95)	92.0 (126)	**<0.0001**
**How GDM is initially treated after diagnosis?**			
Dietary change and increasing physical activity	59.4 (82)	78.3 (108)	**<0.0001**
Insulin is only started if dietary change and physical activity is insufficient	58.0 (80)	64.5 (89)	0.253
**Which food products do you have to restrict if you have GDM?**			
Pie	89.9 (124)	96.4 (133)	**0.022**
Fruits	18.8 (26)	55.8 (77)	**<0.0001**
Sugared soda	94.9 (131)	98.6 (136)	0.063
Fruit juice	77.5 (107)	93.5 (129)	**<0.0001**
**Which fasting blood sugar level is normal in the morning?**			
< 95 mg/dl	42.0 (58)	97.8 (135)	**<0.0001**
**Which blood sugar level is normal 2 hours after eating?**			
< 120 mg/dl	26.8 (37)	96.4 (133)	**<0.0001**
**How can best be checked if your blood sugar levels are sufficiently under control?**			
Based on a finger prick with a glucometer	80.1 (109)	98.5 (134)	**0.0005**
**What do you think about the treatment with insulin for GDM?**			
This can lower the risk of an overweight baby	47.8 (65)	71.3 (97)	**<0.0001**
**What do you think about breastfeeding after a pregnancy with GDM?**			
This is good for the general health of the baby	61.6 (85)	92.0 (127)	**<0.0001**
This can lower the risk of diabetes and overweight in the baby later on	26.8 (37)	37.0 (51)	**0.018**
**What do you think that happens with your GDM after your delivery?**			
GDM disappears completely but I have a strongly increased risk of 50% to develop T2DM within 10 years	44.2 (61)	82.6 (114)	**<0.0001**

GDM: gestational diabetes mellitus; T2DM: type 2 diabetes mellitus; Statistically significant p-values are in bold. .

Table S4. Comparison of correct responses on the knowledge questionnaire between group and individual education after the first education session.

	group education n = 98 % (n)	individual education n = 74 % (n)	p-value
**When is GDM diagnosed?**			
24 - 28 weeks	99.0 (97)	98.6 (73)	1.000
**How is GDM diagnosed?**			
Based on a fasting blood collection in combination with drinking a sugar solution	87.8 (86)	90.5 (67)	0.740
**It's more likely to develop gestational diabetes if you:**			
Are overweight before pregnancy	91.8 (90)	83.8 (62)	0.164
Gain too much weight during the pregnancy	38.8 (38)	36.5 (27)	0.883
Have had GDM during a previous pregnancy	77.6 (76)	70.3 (52)	0.364
Have a first degree relative with diabetes	89.8 (88)	79.7 (59)	0.102
Your age is > 30 years	62.2 (61)	52.7 (39)	0.271
**What are the consequences for the baby if the treatment of GDM is insufficient?**			
Too high birth weight of the baby	95.9 (94)	95.9 (71)	1.000
Increased risk of diabetes for the baby later on	80.6 (79)	77.0 (57)	0.702
Increased risk of overweight for the baby later on	73.5 (72)	75.7 (56)	0.879
**What are the risks for you if the treatment of GDM is insufficient?**			
An increased risk for a difficult delivery	91.8 (90)	90.5 (67)	0.980
An increased risk for preeclampsia	92.9 (91)	86.5 (64)	0.259
An increased risk for a cesarean section	90.8 (89)	91.9 (68)	1.000
**How is GDM initially treated after diagnosis?**			
Dietary change and increasing physical activity	81.6 (80)	77.0 (57)	0.581
Insulin is only started if dietary change and physical activity is insufficient	74.5 (73)	62.2 (46)	0.117
**Which food products do you have to restrict if you have GDM?**			
Pie	96.9 (96)	95.9 (71)	1.000
Fruits	63.6 (62)	55.4 (41)	0.377
Sugared soda	95.9 (94)	94.6 (70)	0.404
Fruit juice	98.0 (96)	95.9 (71)	1.000
**Which fasting blood sugar level is normal in the morning?**			
< 95 mg/dl	98.0 (96)	90.5 (67)	**0.040**
**Which blood sugar level is normal 2 hours after eating?**			
< 120 mg/dl	96.9 (96)	91.9 (68)	0.176
**How can best be checked if your blood sugar levels are sufficiently under control?**			
Based on a finger prick with a glucometer	94.9 (93)	94.6 (70)	1.000
**What do you think about the treatment with insulin for GDM?**			
This can lower the risk of an overweight baby	77.6 (76)	71.6 (53)	0.477
**What do you think about breastfeeding after a pregnancy with GDM?**			
This is good for the general health of the baby	85.7 (84)	95.9 (70)	0.052
This can lower the risk of diabetes and overweight in the baby later on	32.7 (32)	35.6 (26)	0.809
**What do you think that happens with your GDM after your delivery?**			
GDM disappears completely but I have a strongly increased risk of 50% to develop T2DM within 10 years	85.7 (84)	87.8 (65)	0.858

GDM: gestational diabetes mellitus; T2DM: type 2 diabetes mellitus; Statistically significant p-values are in bold. .

Table S5. Satisfaction rates with the given explanation on 12 items after the initial education session for women in group education compared to women in individual education.

	strongly disagree		disagree		neutral		agree		strongly agree		I don't know		p-value
	G % (n)	I % (n)	G % (n)	I % (n)	G % (n)	I % (n)	G % (n)	I % (n)	G % (n)	I % (n)	G % (n)	I % (n)	
**Q1: What is GDM**	0.0 (0)	1.4 (1)	0.0 (0)	0.0 (0)	2.0 (2)	0.0 (0)	21.4 (21)	14.9 (11)	75.5 (74)	83.8 (62)	1.0 (1)	0.0 (0)	0.311
**Q2: Importance of treatment**	0.0 (0)	1.4 (1)	0.0 (0)	0.0 (0)	3.1 (3)	0.0 (0)	16.3 (16)	12.2 (9)	80.6 (79)	86.5 (64)	0.0 (0)	0.0 (0)	0.302
**Q3: Risks for myself**	0.0 (0)	1.4 (1)	1.0 (1)	0.0 (0)	4.1 (4)	1.4 (1)	20.4 (20)	12.3 (9)	73.5 (72)	84.9 (62)	1.0 (1)	0.0 (0)	0.135
**Q4: Risks for my baby**	0.0 (0)	1.4 (1)	0.0 (0)	0.0 (0)	2.0 (2)	0.0 (0)	21.4 (21)	13.5 (10)	75.5 (74)	84.1 (63)	1.0 (1)	0.0 (0)	0.217
**Q5: Treatment with diet**	0.0 (0)	1.4 (1)	0.0 (0)	0.0 (0)	4.1 (4)	1.4 (1)	32.7 (32)	20.3 (15)	63.3 (62)	77.0 (57)	0.0 (0)	0.0 (0)	0.057
**Q6: Treatment with physical activity**	0.0 (0)	1.4 (1)	1.0 (1)	0.0 (0)	3.1 (3)	2.7 (2)	28.6 (28)	24.3 (18)	66.3 (65)	71.6 (53)	1.0 (1)	0.0 (0)	0.643
**Q7: Weight gain**	0.0 (0)	2.7 (2)	2.0 (2)	0.0 (0)	12.2 (12)	4.1 (3)	29.6 (29)	34.2 (25)	55.1 (54)	58.9 (43)	1.0 (1)	0.0 (0)	0.550
**Q8: Measuring blood sugar levels**	0.0 (0)	1.4 (1)	1.0 (1)	0.0 (0)	1.0 (1)	0.0 (0)	17.3 (17)	15.3 (11)	79.6 (78)	83.3 (60)	1.0 (1)	0.0 (0)	0.754
**Q9: Treatment with insulin**	0.0 (0)	1.4 (1)	3.1 (3)	5.6 (4)	23.7 (23)	12.5 (9)	34.0 (33)	38.9 (28)	37.1 (36)	38.9 (28)	2.1 (2)	2.8 (2)	0.539
**Q10: Follow-up after delivery**	0.0 (0)	1.4 (1)	1.0 (1)	0.0 (0)	2.0 (2)	1.4 (1)	31.6 (31)	27.0 (20)	64.3 (63)	70.3 (52)	1.0 (1)	0.0 (0)	0.567
**Q11: Risk of diabetes after delivery**	0.0 (0)	1.4 (1)	1.0 (1)	0.0 (0)	4.1 (4)	0.0 (0)	25.5 (25)	24.3 (18)	68.4 (67)	73.0 (54)	1.0 (1)	1.4 (1)	0.403
**Q12: Breastfeeding**	0.0 (0)	2.7 (2)	3.1 (3)	0.0 (0)	3.1 (3)	2.7 (2)	27.6 (27)	21.9 (16)	66.3 (65)	72.6 (53)	0.0 (0)	0.0 (0)	0.406

Q = question; G: group education; I: individual education; GDM: gestational diabetes mellitus; Statistically significant p-values are in bold

Table S6. Agreement with the feasibility of the advice for the management of GDM for women in group education compared to women in individual education.

	strongly disagree		disagree		neutral		agree		strongly agree		I don't know		p-value
	G % (n)	I % (n)	G % (n)	I % (n)	G % (n)	I % (n)	G % (n)	I % (n)	G % (n)	I % (n)	G % (n)	I % (n)	
**Advice about diet**	0.0 (0)	0.0 (0)	0.0 (0)	1.7 (1)	8.5 (6)	6.7 (4)	36.6 (26)	38.3 (23)	54.9 (39)	53.0 (32)	0.0 (0)	0.0 (0)	0.864
**Advice about physical activity**	0.0 (0)	0.0 (0)	5.6 (4)	8.6 (5)	11.1 (8)	10.3 (6)	43.1 (31)	37.9 (22)	40.3 (29)	43.1 (25)	0.0 (0)	0.0 (0)	0.950
**Advice about treatment with insulin***	20.0 (1)	0.0 (0)	0.0 (0)	0.0 (0)	0.0 (0)	20.0 (1)	60.0 (3)	20.0 (1)	0.0 (0)	20.0 (1)	20.0 (1)	40.0 (2)	0.421
**Advice about weight gain**	0.0 (0)	0.0 (0)	1.4 (1)	0.0 (0)	5.8 (4)	13.6 (8)	43.5 (30)	39.0 (23)	49.3 (34)	44.1 (26)	0.0 (0)	3.4 (2)	0.746
**Advice about SMBG**	0.0 (0)	0.0 (0)	0.0 (0)	0.0 (0)	1.4 (1)	1.7 (1)	36.1 (26)	33.9 (20)	62.5 (45)	64.4 (38)	0.0 (0)	0.0 (0)	0.836
**The advice was too strict and I felt starved**	22.5 (16)	15.0 (9)	38.0 (27)	41.7 (25)	18.3 (13)	20.0 (12)	14.1 (10)	10.0 (6)	5.6 (4)	13.3 (8)	1.4 (1)	0.0 (0)	0.377
**I am confident in the given advice**	1.4 (1)	0.0 (0)	0.0 (0)	0.0 (0)	4.2 (3)	1.7 (1)	35.2 (25)	36.7 (22)	59.2 (42)	61.7 (37)	0.0 (0)	0.0 (0)	0.645

* Only those who were treated with insulin and filled in the questionnaire (n = 10); G: group education; I: individual education; GDM: gestational diabetes mellitus; SMBG: self-monitoring of blood glucose; Statistically significant p-values are in bold

Table S7. Emotional status before the initial education session versus after the follow-up session for the complete Dutch cohort.

	**prior to the initial session (n = 128)**	**After the follow-up session (n = 128)**	**p-value**
total score on CES-D questionnaire ≥ 16, %(n)	26.6 (34)	20.3 (26)	0.186
total STAI-6 score [median (IQR)]	12 (7-17)	10 (5-15)	**<0.0001**

CES-D: Center for Epidemiological Studies Depression; STAI: Spielberger State-Trait Anxiety Inventory; Statistically significant p-values are in bold.

Table S8. Emotional status in group versus individual education at the end of the initial education session.

	**general cohort (n = 164)**	**group education (n = 90)**	**individual education (n = 74)**	**p-value**
total score on CES-D questionnaire ≥ 16, %(n)	26.2 (43)	25.6 (23)	27.0 (20)	0.972
total STAI-6 score [median (IQR)]	11 (6-16)	11 (7-15)	11 (6-16)	0.292

CES-D: Center for Epidemiological Studies Depression; STAI: Spielberger State-Trait Anxiety Inventory; Statistically significant p-values are in bold.
